# P21 Healthcare students’ knowledge of antibiotic ineffectiveness in treating viral infections: a systematic review and meta-analysis

**DOI:** 10.1093/jacamr/dlaf118.028

**Published:** 2025-07-14

**Authors:** Asa Auta, Erick Wesley Hedima, Emmanuel O Adewuyi, Shalkur David, Emmanuel Agada David, Enoche Florence Oga, Davies Adeloye, Barry Strickland-Hodge

**Affiliations:** Faculty of Health, Social Care and Medicine, Edge Hill University, Ormskirk, L39 4QP, UK; Department of Clinical Pharmacy and Pharmacy Practice, Faculty of Pharmaceutical Sciences, Gombe State University, Gombe, Nigeria; School of Population Health, Faculty of Health Sciences, Curtin University, Perth, Western Australia, Australia; Department of Clinical Pharmacy and Pharmacy Practice, University of Jos, Jos, Nigeria; Department of Clinical Pharmacy and Pharmacy Practice, Faculty of Pharmaceutical Sciences, Gombe State University, Gombe, Nigeria; School of Pharmacy and Biomedical Sciences, University of Lancashire, Preston, UK; School of Health & Life Sciences, Teesside University, Middlesbrough Tees Valley, TS1 3BX, UK; School of Healthcare, University of Leeds, Leeds, UK

## Abstract

**Background:**

The overuse of antibiotics by healthcare professionals is often associated with a lack of knowledge regarding the rational use of these medications. We synthesized and analysed existing evidence on healthcare students' knowledge of antibiotic ineffectiveness in treating viral infections to provide pooled global and regional estimates.

**Methods:**

The PubMed®, Embase® (via Ovid) and CINAHL (via EBSCO) databases were systematically searched for studies published between 01 January 2014 and 31 December 2024 that reported Healthcare students’ knowledge of antibiotic ineffectiveness in treating viral infections. Pooled estimates and 95% CI of correct knowledge were determined using random-effects meta-analysis.

**Results:**

Of the 9165 articles identified, 86 studies met the inclusion criteria. Most healthcare students correctly understood that antibiotics are ineffective against viruses, with 70.2% (95% CI: 65.6–74.8) demonstrating this understanding. Only 58.0% (95% CI: 51.4–64.6) knew that antibiotics are ineffective against colds and flu. There were no significant regional variations in the understanding that antibiotics are ineffective against viruses. However, notable differences were evident at the country level. Thailand (30.3%, 95CI: 23.8–37.8), Turkey (35.8%, 95% CI: 32.9–38.8), Mali (39.9%, 95% CI: 35.5–44.5), and China (45.7%, 95% CI: 43.5–47.9) demonstrated lower levels of knowledge, whereas Poland (94.0%, 95% CI: 90.4–97.5), the United States (98.0%, 95% CI: 94.5–99.3), and the United Kingdom (98.4%, 95% CI: 97.2–99.6) exhibited higher levels of understanding.

**Conclusions:**

Our findings indicate significant knowledge gaps in the understanding of the ineffectiveness of antibiotics against viruses in many countries. These knowledge gaps have important implications for the rational use of antibiotics and the prevention of resistance.

